# Prediction of particles deposition in a dilute quasi-steady gravity current by Lagrangian markers: effect of shear-induced lift force

**DOI:** 10.1038/s41598-020-73504-3

**Published:** 2020-10-07

**Authors:** Ali Koohandaz, Ehsan Khavasi, Arameh Eyvazian, Hamid Yousefi

**Affiliations:** 1 Department of Mechanical Engineering, University Of Zanjan, Zanjan, Iran; 2 Institute of Research and Development, Duy Tan University, Da Nang, 550000 Vietnam; 3 Faculty of Electrical – Electronic Engineering, Duy Tan University, Da Nang, 550000 Vietnam; 4 Department of Mechanical Engineering, Amirkabir University Of Technology, Tehran, Iran

**Keywords:** Environmental sciences, Civil engineering

## Abstract

A gravity current in a channel at the presence of a triangular obstacle was investigated using LES simulation and the Eulerian approach. The Saffman–Mei equation was also applied to examine the effect of shear-induced lift force on particle deposition. To this end, particles were considered as Lagrangian markers and injected into gravity current. It is important to keep in mind that the interaction between the gravity current and particles was treated as a one-way coupling. The results show that shear-induced lift force prevents particles to deposit at the entrance of channel, where the velocity gradient is high. Furthermore, a reduction in the rate of sediment deposition can be seen again in the vicinity of obstacle due to high velocity gradient. The important result is that the shear-induced lift force has an important role in the cases with considerable velocity gradient in quasi-steady flows and this force can affect the pattern of sedimentation over time. Q criterion is utilized to depict the vortical structures of flow. Vortical structures with larger diameter, that indicate stronger vortexes, has been seen in various sections of channel, especially in the region near the obstacle due to the presence of obstacle.

## Introduction

Gravity currents are formed when a heavier fluid propagates into a lighter ambient fluid as a result of horizontal hydrostatic pressure gradients^1–3^. A homogeneous current is driven by compositional or temperature differences and a particle-driven current is driven by suspended particles^4^. Continuous particle-driven gravity currents frequently occur in natural settings for instance, seafloor turbidity currents, discharge of particles to the ocean by particle-laden rivers and lava flows^5–10^ and in industrial settings for example in the oil and gas industry and water treatment facilities^11^. They play an important role in the transport of sediments in oceans or lakes^12^. Due to the unpredictable and catastrophic feature of turbidity currents in the nature that may lead to an uncontrollable condition, huge efforts have been aimed at understanding their dynamics in laboratory-scale using either flat bottom boundaries or topographical features^13–23^. The improvement of computational methods and computers over recent years has enabled investigators to conduct fully three-dimensional, depth-resolved simulations of turbidity currents^24–35^.

Noteworthy, the Eulerian approach has been mostly employed in all cases to simulate gravity current which can be assigned to its low computational cost and the acceptable accuracy. Nevertheless, in the cases with a high discrepancy in the concentration, the Lagrangian approach should be used due to the high number of particles. However, the large computational time is the most important constraint of this approach. Exact results can be obtained by injecting Lagrangian markers into a gravity current and tracking them to determine their behavior in the gravity current. In this paper, particles have been injected into the gravity current as Lagrangian markers to investigate their behavior in the flow without considering their effects on the flow (due to low particle loading).

On the other hand, the movement of particles in gravity currents is the result of interactions occurring between several forces such as gravity, aerodynamic drag, pressure gradient, Brownian motion, and an additional force that acts on the particle, known as the shear-induced lift force. Shear-induced lift force has been taken into account in some studies. Many authors have included the shear-induced lift force into their simulations, for example^36–41^. The gravity current was considered as the lock-exchange type and numerically simulated with the Eulerian–Eulerian approach^42^.

In this paper, the Saffman–Mei model was used to evaluate the effect of the shear-induced lift force on particle deposition in gravity currents. By comparing the results with a case without applying lift force to particles, the effect of this force on particles in a dilute quasi-steady gravity current will be clarified. It should be mentioned that the Brownian motion force is important for particle diameter smaller than 0.5 μm hence this force was neglected in this study as the particle diameter ranged from 12 to 200 μm. It is worth noting that a stochastic dispersion method was adopted for applying velocity fluctuations generated by small eddies (unresolved turbulence) on the particles to better predict the particle dispersion in LES of a turbulent flow.

In the present study Large Eddy Simulation (LES) was employed using a computational code in OpenFOAM software to simulate a turbulent gravity current. Moreover, a computational code was used in Java software for analyzing the particle deposition behavior. In “Methodology” section governing equations and modeling approaches are presented. “Results and discussions” section is focused on discussion and comparison of the numerical results and finally, conclusions of the current investigation will be expressed in “Conclusion” section.

## Methodology

### Governing equations

**Figure 1:**
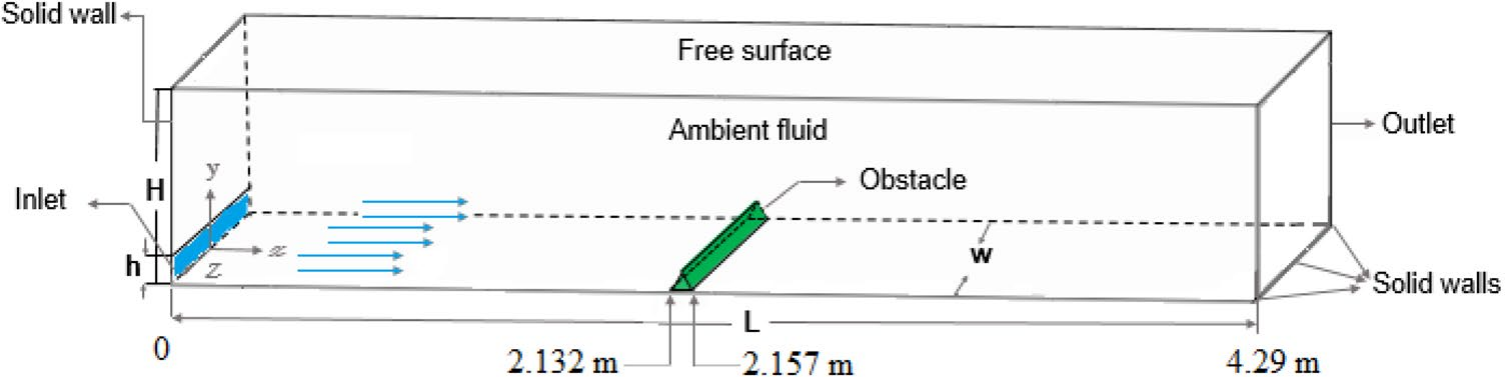
Schematic of a three-dimensional channel with an obstacle in the middle of it.

**Figure 2:**
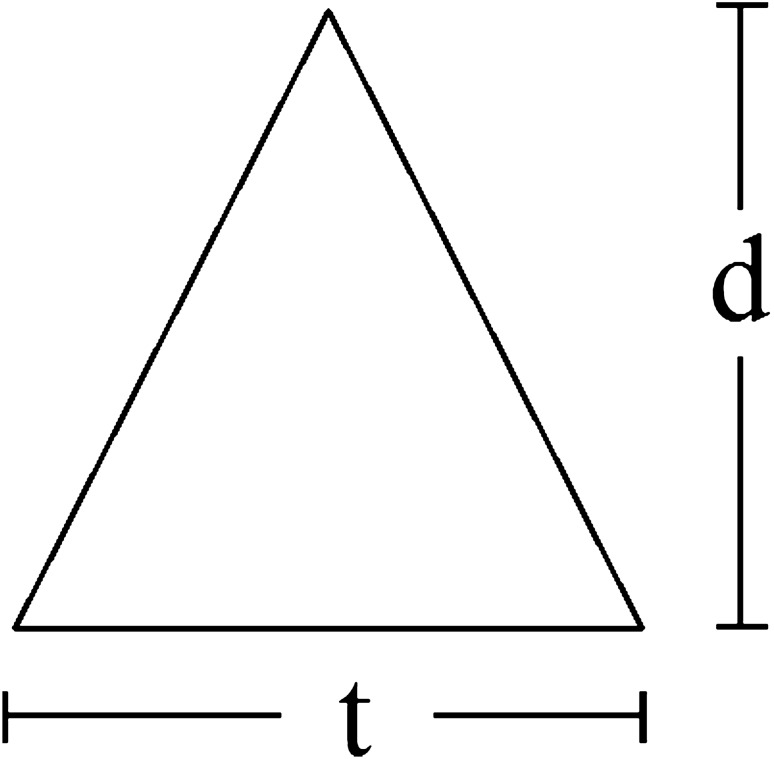
Schematic of a triangular obstacle.

This study is focused on a quasi-steady gravity current in a 3D channel with a triangular obstacle, as shown in Figs. 1 and 2. The dimensions of the channel and obstacle are presented in Tables 1 and 2, respectively.

**Table 1:** Dimensions of the channel.

Parameters	H	h	L	W
Values (cm)	30	3	429	30

**Table 2:** Dimensions and location of the obstacle.

Parameters	t (cm)	d (cm)	Location of the first point in the channel	Location of the second point in the channel
Rectangular obstacle	2.58	5.58	2.132 m	2.1578 m

Based on Boussinesq approximation a set of equations can be simplified by assuming a constant density equal to the reference density $\rho_{0}$, except in the gravity-buoyancy term of the momentum equations, if the difference of the current density with the ambient fluid is sufficiently low^34,35,43–46^.

Thus, the Navier–Stokes equations in Boussinesq approximation was employed to model the flow motion, along with a transport equation for the Eulerian description of the concentration field:

1 $$ \frac{{\partial u_{k} }}{{\partial x_{k} }} = 0 $$

2 $$ \frac{{\partial u_{l} }}{\partial t} + u_{k} \frac{{\partial u_{l} }}{{\partial x_{k} }} = \upsilon_{w} \frac{{\partial^{2} u_{l} }}{{\partial x_{k} \partial x_{k} }} + \frac{\partial }{{\partial x_{k} }}\left( {2\upsilon_{SGS} S_{lk} } \right) - \frac{1}{{\rho_{w} }}\frac{{\partial_{p} }}{{\partial x_{l} }} - g^{^{\prime}} c\delta_{21} $$

3 $$ \frac{{\partial c_{n} }}{\partial t} + u_{k} \frac{{\partial c_{n} }}{{\partial x_{k} }} = \alpha \frac{{\partial^{2} c_{n} }}{{\partial x_{k} \partial x_{k} }} + \frac{\partial }{{\partial x_{k} }}\left( {\alpha_{SGS} \frac{{\partial c_{n} }}{{\partial x_{k} }}} \right)  $$

Here $\rho_{W}$ and $\nu_{w}$ are the density and kinematic viscosity of ambient flow, respectively. $g^{\prime }$ shows the reduced gravity acceleration $g^{\prime } = \beta g$ where $\beta = {{(\rho - \rho_{w} )} \mathord{\left/ {\vphantom {{(\rho - \rho_{w} )} {\rho_{w} }}} \right. \kern-\nulldelimiterspace} {\rho_{w} }}$; moreover, $C = {{(\rho - \rho_{w} )} \mathord{\left/ {\vphantom {{(\rho - \rho_{w} )} {(\rho_{\max } - \rho_{w} )}}} \right. \kern-\nulldelimiterspace} {(\rho_{\max } - \rho_{w} )}}$ where $\rho$ is the density of current. The Schmidt number $Sc = {\nu \mathord{\left/ {\vphantom {\nu \alpha }} \right. \kern-\nulldelimiterspace} \alpha }$ refers to the ratio of the kinematic viscosity $\nu_{w}$ to the mass diffusivity $\alpha$. Considering previous studies^43,47^, the properties of the current are insensitive to the Schmidt number when $Sc \ge 1$ hence its value was considered $Sc = 1$.

It should be mentioned that subgrid-scale eddy viscosity, $\nu_{SGS}$, is modeled by means of the large eddy simulation method, thus based on turbulent Schmidt number $Sc_{SGS} = 1$^48,49^, $\alpha_{SGS}$ will be computed through the equation:

4 $$ \alpha_{SGS} = \frac{{\upsilon_{SGS} }}{{Sc_{SGS} }} $$

It is worth mentioning that in this paper a dilute density current was studied (e.g. saline water or a dilute turbidity current) where the flow is not affected by the presence of particles. Based on this approach, sediment fall velocity does not affect the flow behavior, which should be taken into consideration when there is a significant discrepancy between the density of the gravity current and that of ambient fluid (a strong density current). Since the purpose of this study was to predict the fate of particles in a dilute density current, sediment fall velocity was not considered in the concentration equation. This investigation only considered sphere particles. The particle diameter ranges from 12 to 200 μm, distributed according to the Rosin–Rammler distribution function. The particle density was 1350 kg/m^3^ in all runs.

In the Lagrangian approach, the trajectory of each particle is computed by solving the momentum equation based on Newton’s second law:

5 $$ \frac{{d(m_{p} v_{i} )}}{dt} = \sum {F_{i} } $$

In this study, gravity force, drag force, pressure gradient force and shear-induced lift force, are taken into consideration for each particle injected into the flow:

6 $$ \ddot{\vec{r}} = \frac{{(\vec{f}_{sa} + \vec{f}_{D} + \vec{f}_{P} + \vec{f}_{g} )}}{m} $$

Drag force is quantified by the drag coefficient, C_D_, through the following equation:

7 $$ \vec{f}_{D} = - C_{D} \frac{\pi }{8}\rho d_{p}^{2} \left| {\vec{u} - \vec{v}} \right|(v_{i} - u_{i} ) $$

8 $$ C_{D} = \max (0.44, C_{D} \,{\text{of Eq}}{. }10) $$

9 $$ C_{D} = \frac{{24.0(1.0 + 0.15{\text{Re}}_{p}^{0.687} )}}{{{\text{Re}}_{p} }} $$

where ${\text{Re}}_{p}$ is the particle Reynolds number:

10 $$ {\text{Re}}_{p} = \frac{{\left| {\vec{u} - \vec{v}} \right|d_{p} }}{v} $$

The gravity force is expressed as:

11 $$ f_{{gravity_{i} }} = (\rho_{p} - \rho )\frac{\pi }{6}d_{p}^{3} g_{i} $$

For calculating the lift force on spherical solid particles, the Saffman–Mei model^50^ was found to be suitable. It is a generalization of the older Saffman model^51^, which can be applied to a lower range of particle Reynolds numbers than the Saffman–Mei model.

Shear-induced lift force can be expressed by Lamb vector^42,52^:

12 $$ \vec{f}_{LS} = C_{L} \rho_{f} V_{p} \vec{\omega }_{f} \times \vec{u}_{r} $$

where

13 $$ \vec{u}_{r} = \vec{u}_{P} - \vec{u}_{f} $$

And:

14 $$ C_{L} = \frac{3}{4}C_{LA} \frac{{{\text{Re}}_{s} }}{{{\text{Re}}_{\omega } }} $$

Based on the Saffman–Mei model, $C_{LA}$ is:

15 $$ \frac{{C_{LA} }}{{C_{LA,Saffman} }} = \left\{ {\begin{array}{*{20}l} {f({\text{Re}}_{s} ,{\text{Re}}_{\omega } ),} \hfill & {0 \ll {\text{Re}}_{s} \ll 40} \hfill \\ {0.0524(\alpha_{s} {\text{Re}}_{s} )^{1/2} ,} \hfill & {40 \ll {\text{Re}}_{s} \ll 100} \hfill \\ \end{array} } \right. $$

where

16 $$ C_{LA,Saffman} = \frac{{2(C_{L}^{\prime } )}}{\pi }\epsilon_{s} \quad {\text{and}}\quad \epsilon_{s} = \frac{{\sqrt {{\text{Re}}_{\omega } } }}{{{\text{Re}}_{s} }}\quad C_{L}^{\prime } = 6.46 $$

The function $f({\text{Re}}_{s} ,{\text{Re}}_{\omega } )$ is:

17 $$ f({\text{Re}}_{s} ,{\text{Re}}_{\omega } ) = (1 - 0.3315\alpha_{s}^{{{1 \mathord{\left/ {\vphantom {1 2}} \right. \kern-\nulldelimiterspace} 2}}} )e^{{ - 0.1{\text{Re}}_{p} }} + 0.3315\alpha_{s}^{{{1 \mathord{\left/ {\vphantom {1 2}} \right. \kern-\nulldelimiterspace} 2}}} $$

where

18 $$ \alpha_{s} = \frac{1}{2}{\text{Re}}_{s} \epsilon_{s}^{2} = \frac{1}{2}\frac{{{\text{Re}}_{\omega } }}{{{\text{Re}}_{s} }} $$

And finally, the pressure gradient force is:

19 $$ \vec{f}_{p} = \frac{{\rho_{f} }}{{\rho_{p} }}\vec{u}_{p} .\nabla \vec{u}_{f} $$

### Numerical methods

The computational grid included 1108 × 38 × 30 cells, in the streamwise, bottom wall-normal, and spanwise directions, respectively.

According to Pelmard et al.^43^, more than 16 cells in the geometry are usually used to be a minimum for an accurate LES and to represent the largest structures. Thus the maximum mesh size can be scaled with *H*/16 where *H* is the height of the current. On the other hand, the minimum mesh size of an LES should be higher than the Kolmogorov length scale, which is expressed according to the energy cascade theory of homogeneous turbulence, as $( H/2){\text{Re}}^{-3/4}$. Consequently, the mesh size h should be $\left( {{H \mathord{\left/ {\vphantom {H 2}} \right. \kern-\nulldelimiterspace} 2}} \right){\text{Re}}^{{{{ - 3} \mathord{\left/ {\vphantom {{ - 3} 4}} \right. \kern-\nulldelimiterspace} 4}}} \le h < {H \mathord{\left/ {\vphantom {H {16}}} \right. \kern-\nulldelimiterspace} {16}}$^43^.

According to the structure of the quasi-steady flow in present simulation, H as the height of the current is nearly 0.1 m (see Fig. 7g), so the mesh size will be less than 0.00625 m for capturing the gravity current.

$y^{ + }$ is defined as the dimensionless distance from the wall and its value should be sufficient to capture the viscous sub-layer^43^:

20 $$ y^{ + } = \frac{{u_{\tau } \Delta y}}{\upsilon } $$

where

21 $$ u_{\tau } = \sqrt {\frac{{\tau_{w} }}{\rho }} $$

In this paper, near the bottom surface $y^{ + }$ was approximately equal to one, and it was sufficient to resolve the viscous sub-layer, and avoiding the 3 of wall layer models^53,54^.

The velocity of gravity current and subsequently the velocity of injected particles at the entrance of the channel was 0.11 m/s, the inlet concentration had a uniform value of 0.9 while the outlet pressure was zero. Due to atmosphere conditions out of the channel, the no-slip condition was considered at the walls (sidewalls and bottom wall), and the symmetry boundary condition is used for the top surface of the domain. This type of boundary condition is used to apply mirror-symmetry conditions in simulations and described by removal of the tangential stress, it means the fluxes across the symmetry are zero as well as the normal components of all variables, but flow is free to slide in tangential directions. According to our assumption, at upper surface momentum transport does not occur and we do not expect considerable waves (the water surface in a windless condition in laboratory). So this type of boundary condition does not affect the behavior of the gravity current unless the symmetry plane is very close to the interest region, consequently, it can be assumed like a surface without momentum transport. Injected particles considered to be non-cohesive. Elghobashi^55^ stated that for very low values of $(\phi_{p} ( \le 10^{ - 6} )$, the particles do not have any considerable effect on the turbulence, and the interaction between the particles and turbulence should be one-way coupling.

22 $$ \phi_{p} = \frac{{MV_{P} }}{V} $$

Here, *V* denotes the volume occupied by fluid and particle, $V_{P}$ is the volume of the particle and *M* represents the number of particles. Based on Elghobashi^55^, in the regimes where $10^{ - 6} < \phi_{p} \le 10^{ - 3}$ the two-way coupling should be considered because particle loading is large enough to affect the turbulence structure. Moreover, in the regions where $10^{ - 3} < \phi_{p}$, the particle/particle collision should be considered in addition to the two-way coupling between particles and turbulence, which is termed as four-way coupling. Consequently, in the present study, the interaction between the gravity current and particles was treated as a one-way coupling, assuming that the effect of particles on the turbulent flow is negligible due to low particles loading. In other words, the value of particle volume fraction (calculated by Eq. 5) is less than $10^{ - 6}$, therefore, the effect of turbulent flow on the particles is considerable, while the effect of particles on the behavior of turbulent flow is negligible^55^. Since the interaction between particles and flow is one-way coupling due to low particles loading, there is no interaction between particles; so they are unable to accumulate. In order to discretize the divergence terms in equations, Gauss linear method, Gauss QUICK method, Gauss limited linear method and Gauss LUST method were utilized. Furthermore, modified Gauss linear was used to discretize the laplacian terms. QUICK method is a fourth order method for the gradient term a fourth order method presented by Peer et al.^56^ was employed meanwhile time discretization scheme was a backward second order method^30^.

Despite the variations of velocity profiles in the different conditions of gravity currents, it is possible to superimpose the dimensionless velocity profiles by use of depth average parameters. In other words, dimensionless velocity profiles are identical and the gravity current is self-similar. It can be concluded that if the results of numerical simulation are accurate, considering gravity current is self-similar, the dimensionless velocity profiles should be superimposed onto each other, and it consequently ensures the accuracy of numerical simulation results. Many researchers have studied this issue^57,58^. To obtain non-dimensional velocity profiles, the values of velocity and the current height should be non-dimensionalized by depth average velocity and height. These parameters are defined based on governing physical laws and relative to the hydraulic of the flow. To extract relations according to average velocity and height, first, the relations of mass and momentum conservation laws should be written. Mass conservation (22) and momentum conservation (23) are calculated for a cross-section of the gravity current:

23 $$ UH = \int\limits_{0}^{\infty } {u\left( y \right)dy} $$

24 $$ U^{2} H = \int\limits_{0}^{\infty } {\left( {u\left( y \right)} \right)^{2} dy} $$

Consequently:

$$ U = \frac{{\int\limits_{0}^{\infty } {\left( {u\left( y \right)} \right)^{2} dy} }}{{\int\limits_{0}^{\infty } {u\left( y \right)dy} }} $$

$$ H = \frac{{\left( {\int\limits_{0}^{\infty } {u\left( y \right)dy} } \right)^{2} }}{{\int\limits_{0}^{\infty } {\left( {u\left( y \right)} \right)^{2} dy} }} $$

In the above relations, u(y) is the streamwise velocity at the height of y from the bed.

### Validation

#### Validation of Lagrangian method with experimental results

**Figure 3:**
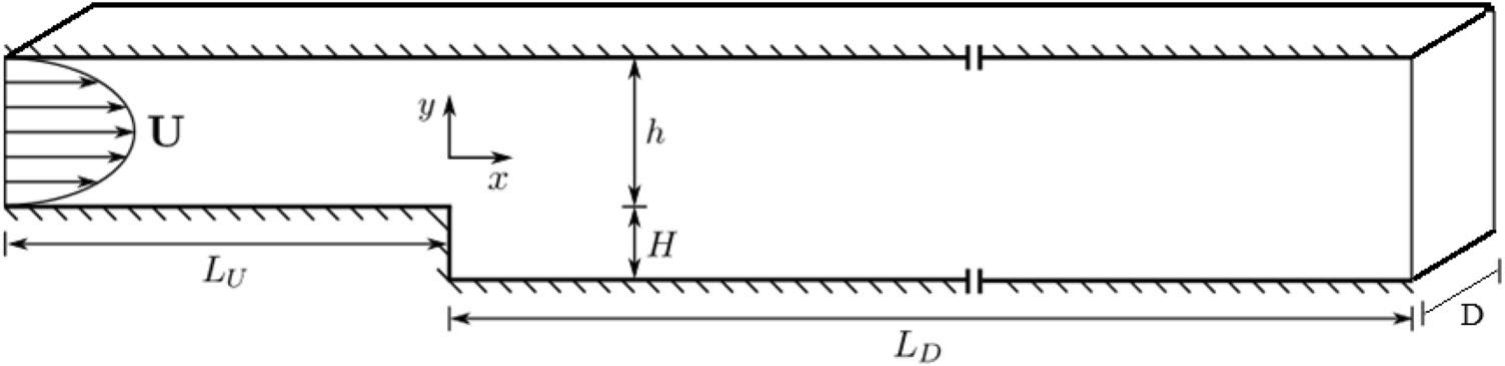
Schematic view of the channel used in the experimental investigation performed by Fessler et al.^59^.

Experimental results obtained by Fessler et al.^59^ were used to validate the numerical simulation (see Fig. 3).

In the mentioned experimental investigation, a backward-facing step flow with a fully developed flow at the inlet with three types of particles was used. The dimensions of the channel are presented in Table 3.

**Table 3:** Dimensions of the channel used in the experimental investigation by Fessler et al.^59^.

Parameters	H (mm)	h (mm)	L_V_	L_D_	U_0_ (m/s)	D
Values	26.7	40	5 h	35 h	10.5	1

**Figure 4:**
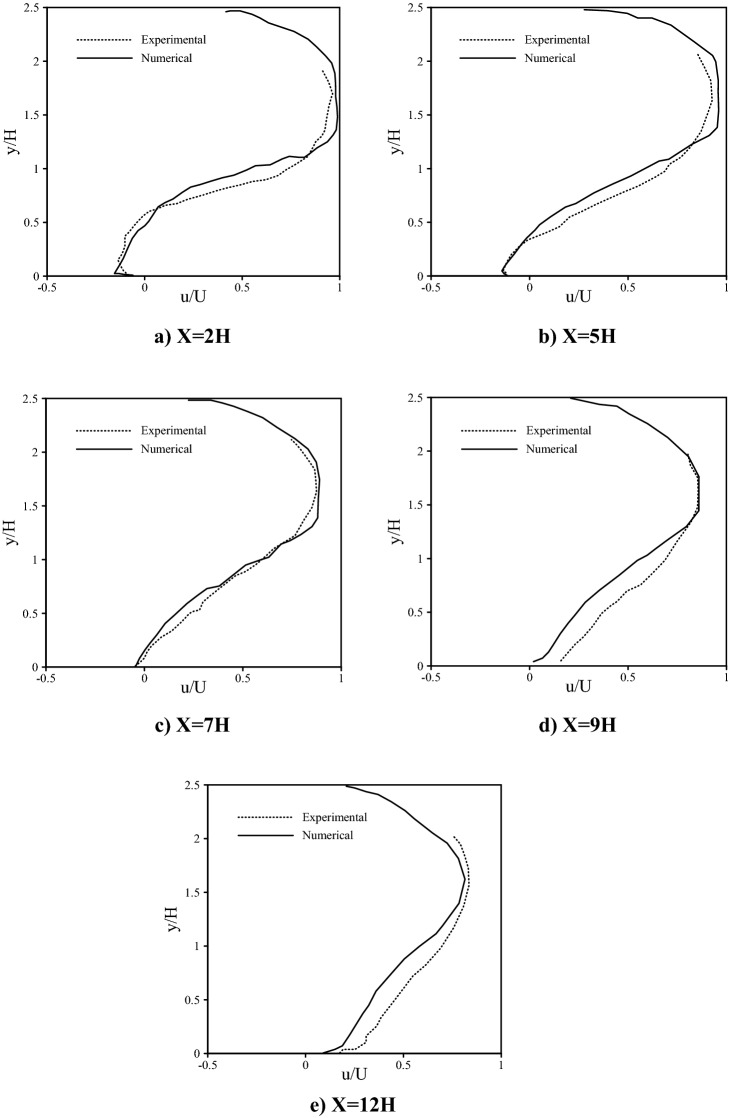
Comparison of velocity profiles obtained from the Eulerian–Lagrangian numerical simulation, with the experimental results of Fessler et al.^59^.

To validate the behavior of Lagrangian particles in this study, an Eulerian–Lagrangian simulation was conducted similar to Fessler et al.^59^ research. The interaction between the current and particles was treated as a two-way coupling because of the high particle loading. Accordingly, velocity profiles obtained from the simulation (which is also affected by the particles) were compared with experimental data of Fessler et al.^59^ in five locations (see Fig. 4). The consistency between the results was acceptable.

#### Validation of Eulerian method

**Figure 5:**
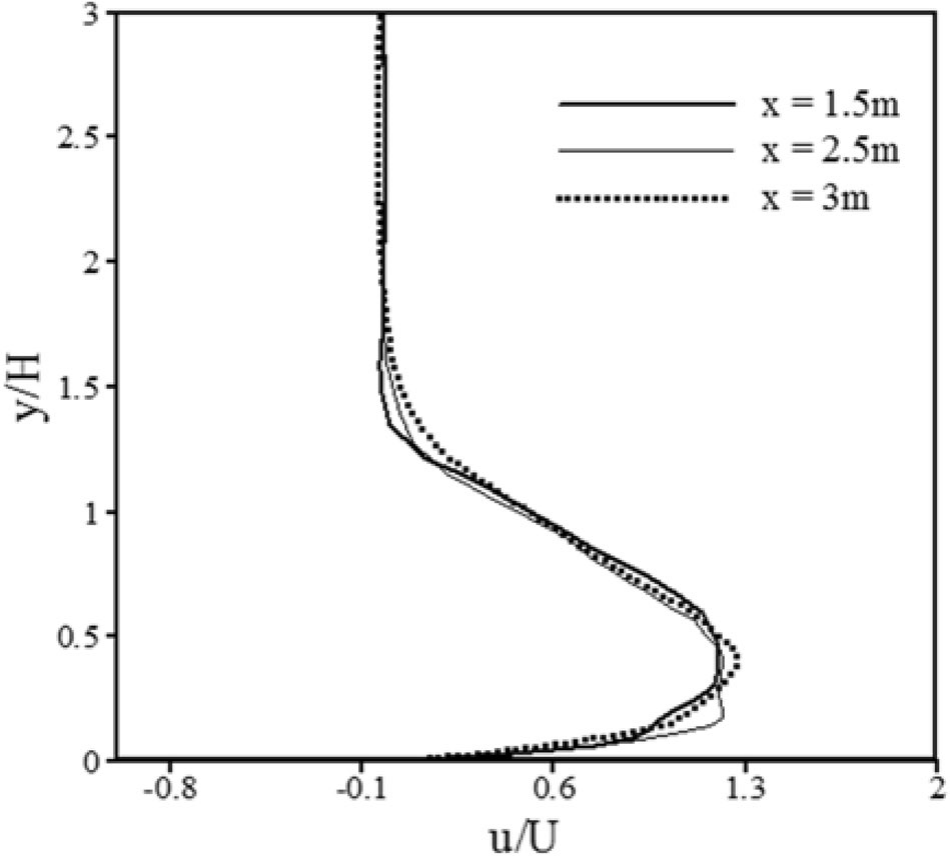
Dimensionless velocity profiles according to the depth average parameters, at various locations (x = 1.5 m, x = 2.5 m, x = 3 m) obtained from the present study.

Altinakar et al.^60^ and Garcia^61^ reported that the maximum values of gravity currents are related to the depth average parameters. For example, according to the experimental results, Altinakar et al.^60^ revealed that the ratio of the maximum velocity to the average velocity is about 1.3 in all the cases and for every location of domain. In this regard, Fig. 5 is presented showing the dimensionless velocity profiles according to the depth average parameters, at various locations (x = 1.5 m, x = 2.5 m, x = 3 m) obtained from the present study.

The values of (u_max_/U) and (y/H), calculated from this study, were compared with the results of Altinakar et al.^60^ and Garcia^61^ as listed in Table 4.

**Table 4:** Comparison of the ratio of (u_max_/U) and (y/H) calculated from this study, with the results of Altinakar et al.^60^ and Garcia^61^.

Parameters	u_max_/U	y/H	% difference between experimental and present numerical results (u_max_/U)	% difference between experimental and present numerical results (y/H)
Altinakar et al.^60^	1.3	0.3	5.8%	12.1%
Garcia^61^				
Results of the present investigation	1.2284	0.3413		

#### Comparison between concentration profile and particles profile

**Figure 6:**
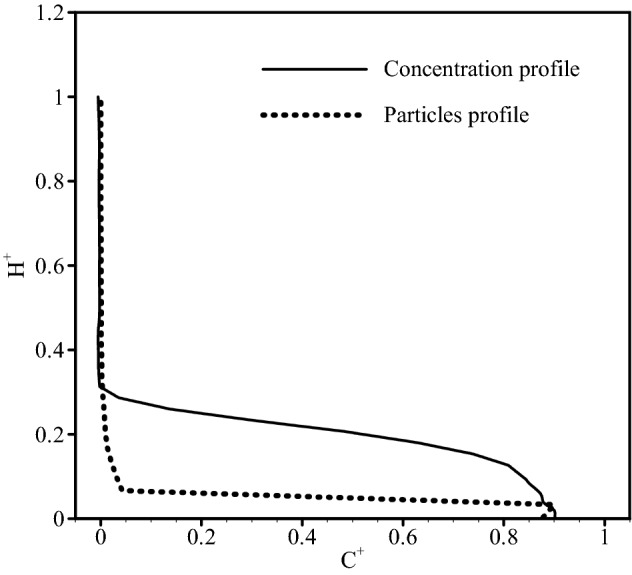
Comparison between concentration profile and particle profile at the position x = 1 m after 150 s.

To show an acceptable agreement between the concentration profiles and those implied by the transported particles, Fig. 6 is presented. This figure compares the particle profile and concentration profile computed from the Eulerian flow field at the position x = 1 m after 150 s. $H^{ + }$ and $C^{ + }$ are respectively the dimensionless channel height and concentration parameter.

The difference between the two profiles in Fig. 6 is reasonable because the gravity force was taken into account for each particle injected to the flow and this force derived the particles toward the bottom.

## Results and discussions

### The effect of shear-induced lift force on sediment deposition

**Figure 7:**
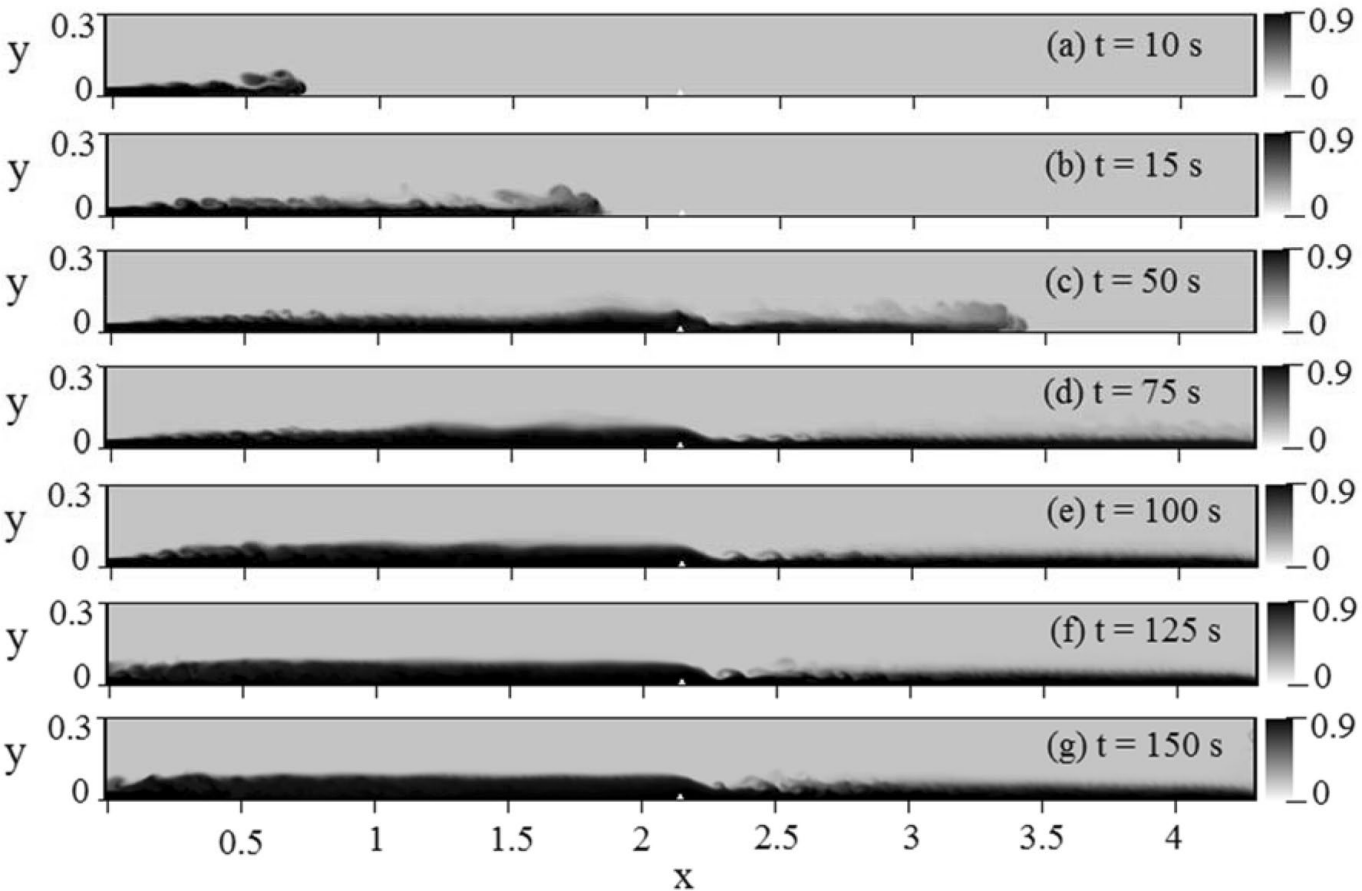
Temporal evolution of the gravity current in the channel in different seconds.

The effect of shear-induced lift force was investigated to predict the sediment deposition in a channel with a triangular obstacle and a height of 2.58 cm. Figure 7 depicts the gradual propagation of the gravity current in the channel. Results were investigated 150 s after the entrance of the gravity current when the flow reached a quasi-steady state (see Fig. 7g). It is worth mentioning that a quasi-steady state is a state in which meanflow properties do not change with time. In other words, the time-average of flow quantities at each selected location in channel is independent of time for example mean velocity remains unchanged regardless of fluctuations caused by turbulence, hence, it is a state that the production of turbulent kinetic energy from the meanflow (the energy associated with the large eddies) is equal with the dissipation of turbulent kinetic energy at the smallest Kolmogorov scale and flow remains temporally invariant.

**Figure 8:**
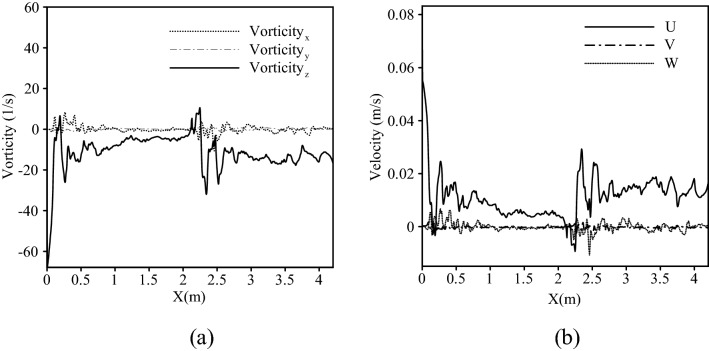
(**a**) Vorticity in three directions (**b**) velocity in three directions along the channel.

It is worth mentioning that according to the Mei–Saffman formulation (12), the magnitude and the direction of shear-induced lift force can change based on the magnitude and the direction of the lamb vector. It should be useful to observe the magnitude of vorticities and velocities in three directions along the channel. Figure 8 shows that vorticity in the spanwise direction where the velocity in streamwise direction showed dominant values in the entrance and vicinity of the obstacle, compared to values of other directions.

**Figure 9:**
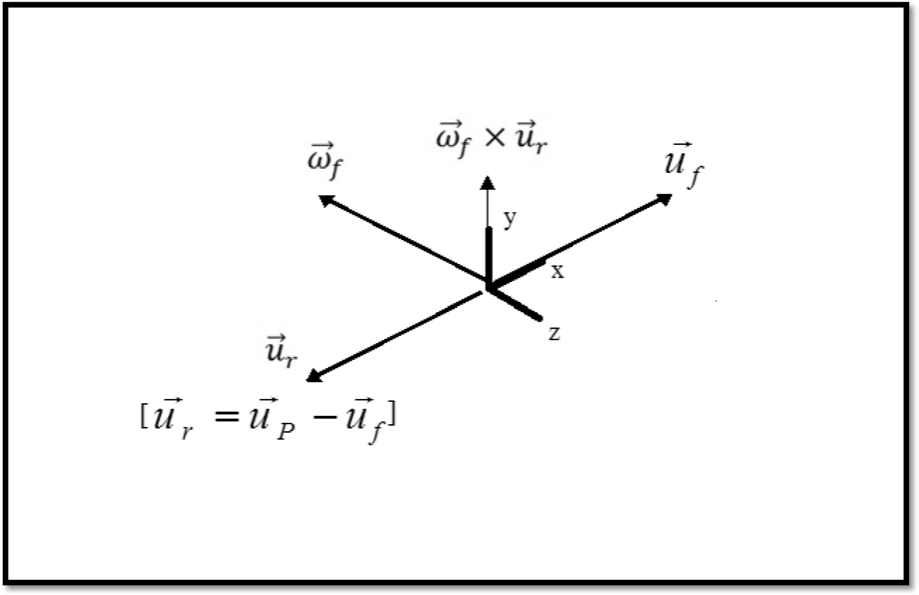
The direction of lamb vector in the entrance of the channel and near the obstacle.

Consequently, it should be expected that the shear-induced lift force acted in an upward direction leading to a decrease in the amount of deposited particles in the aforementioned regions. (See Fig. 9).

**Figure 10:**
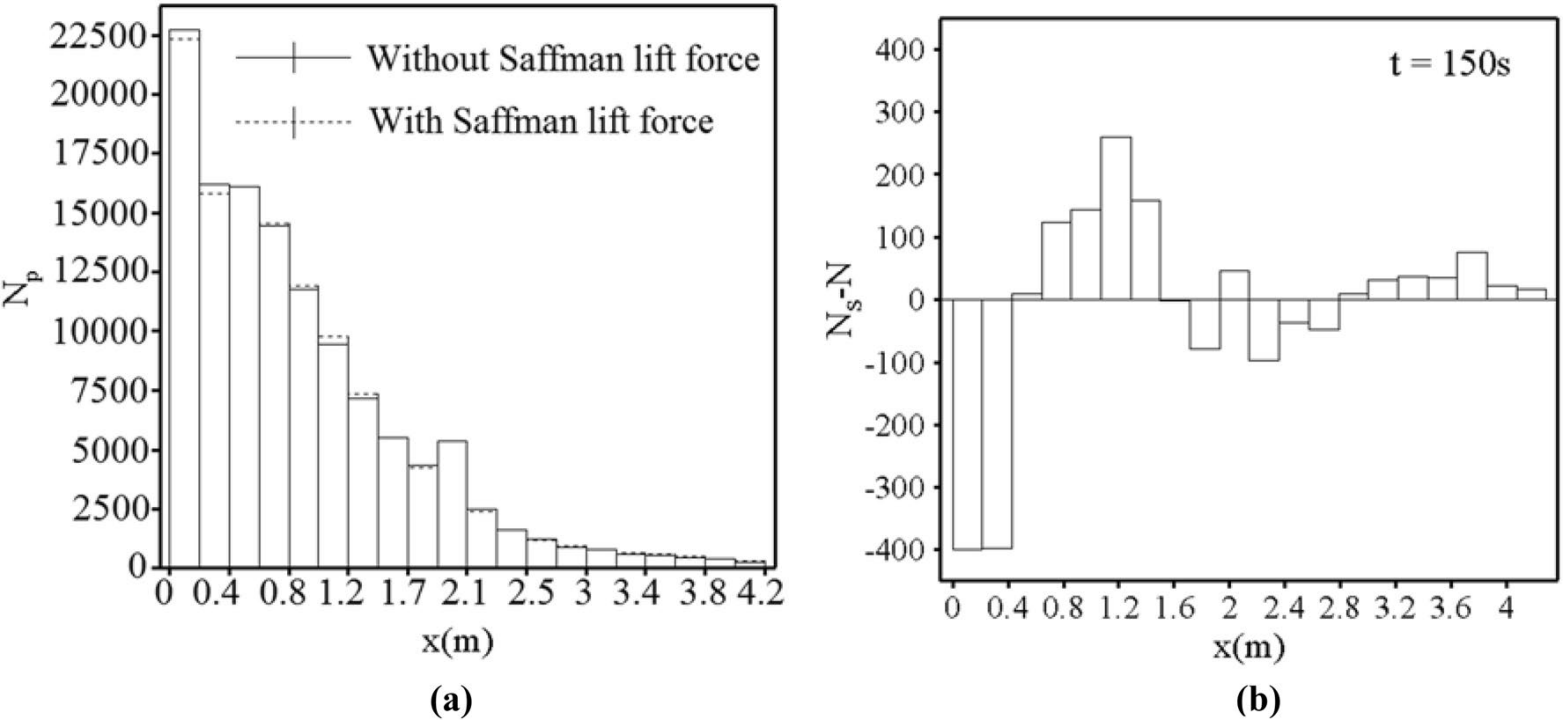
The sedimentation profiles (**a**) Comparison of the number of deposited sediment in the cases with and without the Saffman lift force at 150 s. (**b**) The difference of sediment deposition in the case with and without the Saffman force at 150 s.

The number of particles at the distance of 1 mm from the bottom of the channel, for the cases with and without this force were compared (see Fig. 10a). It should be noted that for the sake of simplicity, the number of particles captured at the distance of 1 mm from the channel bed was expressed and assumed as the amount of sediment deposition. According to Fig. 10a, shear-induced lift force affected the sediment deposition due to high vorticity in the initial section of the channel, thus, the number of deposited particles showed a decrease by 1.75% in the first section of the channel (0–0.2145 m) and 2.44% in the second section (0.2145–0.429 m) at 150 s.

**Figure 11:**
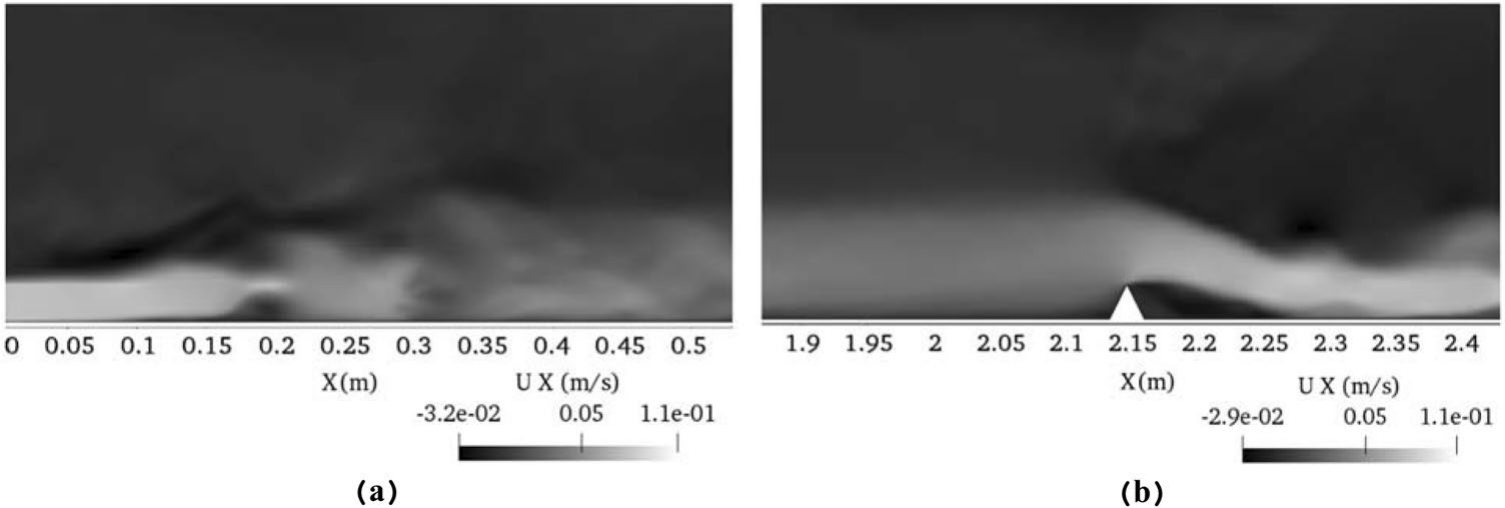
Contours of velocity along the streamwise direction (**a**) At the channel entrance and (**b**) The vicinity of the triangular obstacle.

For the sake of clarity, the difference in the number of deposited particles in the cases with and without Saffman force was plotted at 150 s (see Fig. 10b). In this figure, N_S_ is the number of deposited particles at the presence of shear-induced lift force and N shows the number of deposited particles in the case without applying this force. Negative values can be seen in high-vorticity regions such as the channel entrance and around the obstacle, whereas the positive values were detected in the regions with low values of vorticity. These results confirm the aforementioned statements. In this figure just before and after the obstacle, two humps can be seen, indicating the effect of a triangular obstacle to generate the velocity gradient, and consequently a reduction in the sediment deposition rate in the case where the lift force is applied on the particles. To gain a better insight into the behavior of flow around the obstacle, Fig. 11 is presented. Black regions in Fig. 11 show low values of velocity while white zones are indicative of high-velocity values. White indicators refer to regions with high-velocity gradient and consequently high values of vorticity.

Although with applying the Saffman force the number of deposited sediments decreased in the entrance of the channel, the deposition rate of particles increased at the locations farther than entrance (regions from 0.429 to 1.51 m) where the number of deposited particles is 1.172% more than the case without this force.

### Behavior of vorticity

**Figure 12:**
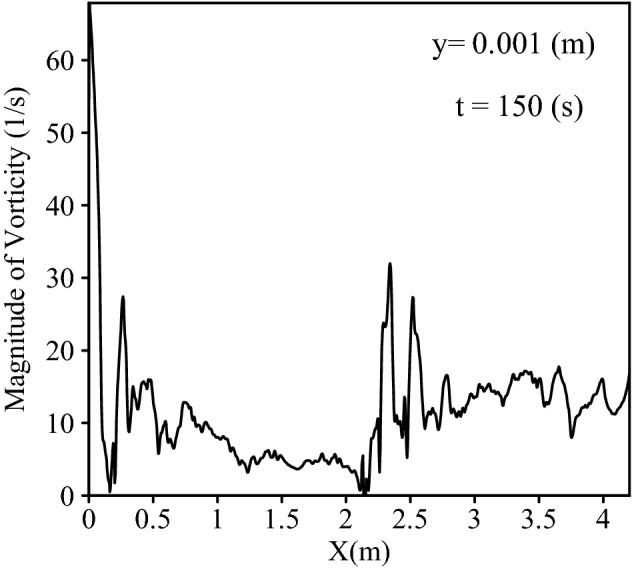
The magnitude of vorticity along the channel at a distance of 1 mm from the bed.

**Figure 13:**
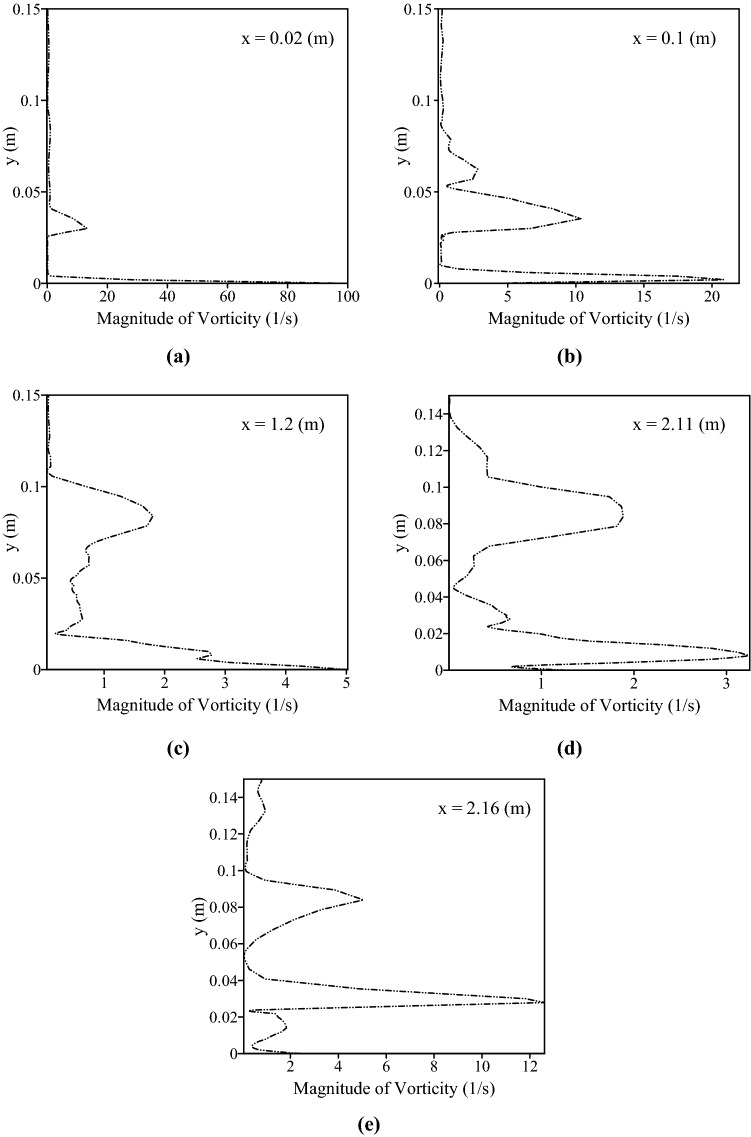
The magnitude of vorticity in the vertical direction in specific locations of channel at the positions (**a**) x = 0.02 m, (**b**) x = 0.1 m (**c**) 1.2 m (**d**) x = 2.11 m (before obstacle), (**e**) x = 2.16 m (after the obstacle), at 150 s.

To investigate the reason for this behavior, the behavior of vorticity was analyzed as presented in Figs. 12 and 13. Figure 12 depicts the values of vorticity over a central line along the channel at a distance of 1 mm from the bed, and Fig. 13 shows the magnitude of vorticity in the vertical direction in some specific locations.

Although the vorticity along the channel showed severe fluctuations, it can be seen that vorticity at the entrance of the channel has high values. For example, in x = 0.00429 m, the vorticity reached a peak with a value of 67.87 1/s, also, Fig. 13a shows the high magnitude of vorticity (100.03 1/s) in the x = 0.02 m. Looking at Fig. 12, it is apparent that the magnitude of vorticity was gradually declined in the areas from approximately x = 0.5 m to areas near the obstacle (x = 2 m). For example, Fig. 13c shows that the vorticity in x = 1.2 m is nearly 5.02 1/s. The particles which were prevented from sedimentation by lift force at the entrance of the channel were transformed by the flow and deposited in regions where the values of vorticity were lower than the entrance. An important point in Fig. 10a,b is the effect of shear-induced lift force on the rate of particle deposition in the areas of nearly 1.9 m to 2.8 m where the presence of obstacle led to the considerable values of vorticity especially after the obstacle. In the case with shear-induced lift force, the amount of deposited particles after the obstacle (areas from 2.14 to 2.78 m) was 3.06% less than another case. This reduction can be explained according to Fig. 12 where the magnitude of vorticity rose and reached 32 1/s in x = 2.34 m Moreover, Fig. 13 shows that in the location after the obstacle (x = 2.16 m) vorticity had a maximum value of 12.61 1/s in y = 0.027 m reflecting the effect of obstacle on the flow structure. Subsequently, particles can deposit in the downstream where the values of vorticity are lower so the number of deposited particles from 2.7 to 4.29 m was 5.39% more in the case with shear-induced lift force as compared with the other case.

### The effect of shear-induced lift force over time

**Figure 14:**
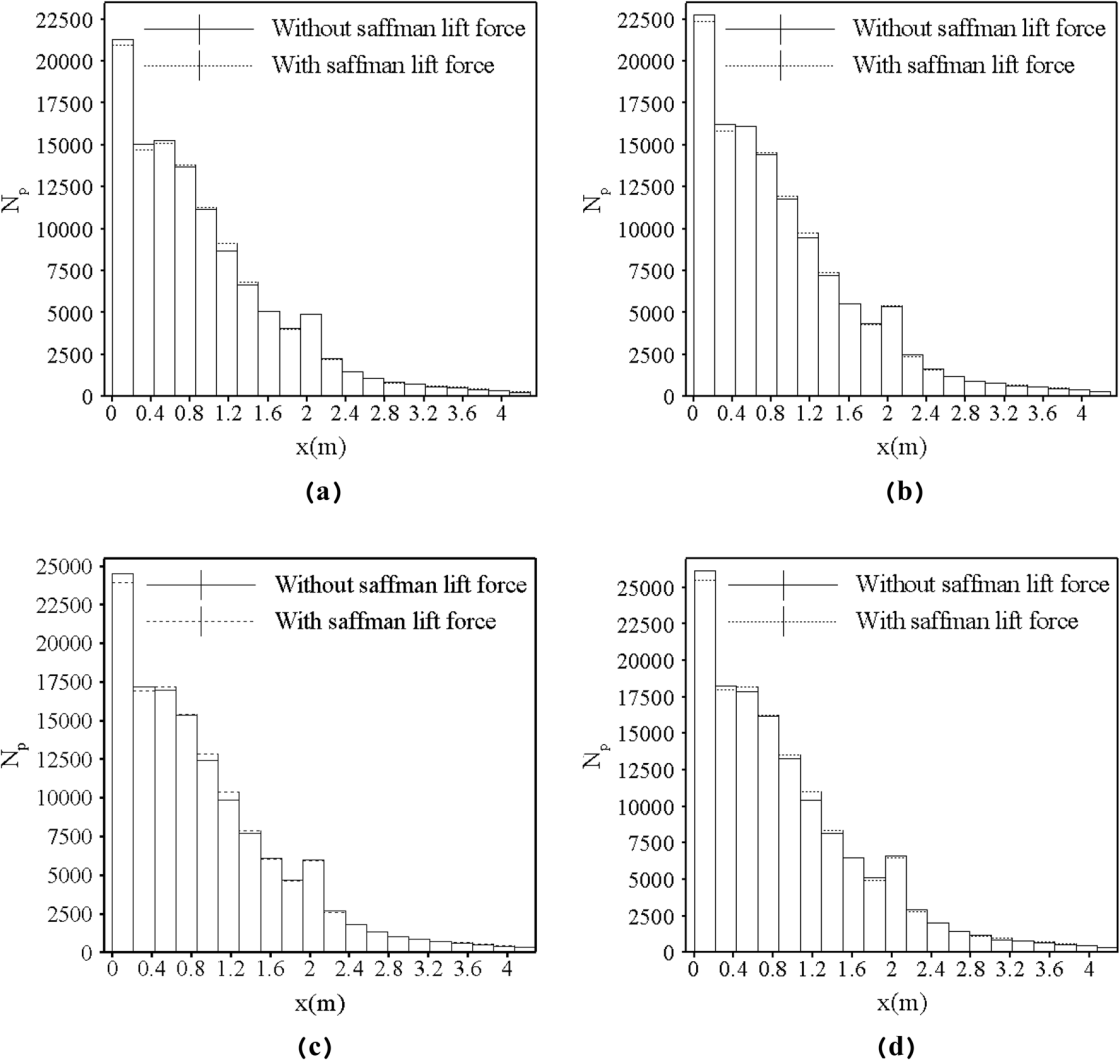
Deposition profiles on the channel bed at the (**a**) 140 s, (**b**) 150 s, (**c**) 160 s, (**d**) 170 s.

Deposition profiles at different times are plotted in Fig. 14. As 1000 particles are injected into the gravity current at every second, it can be expected that the amount of sediment deposition on the channel bed, increases by time.

**Figure 15:**
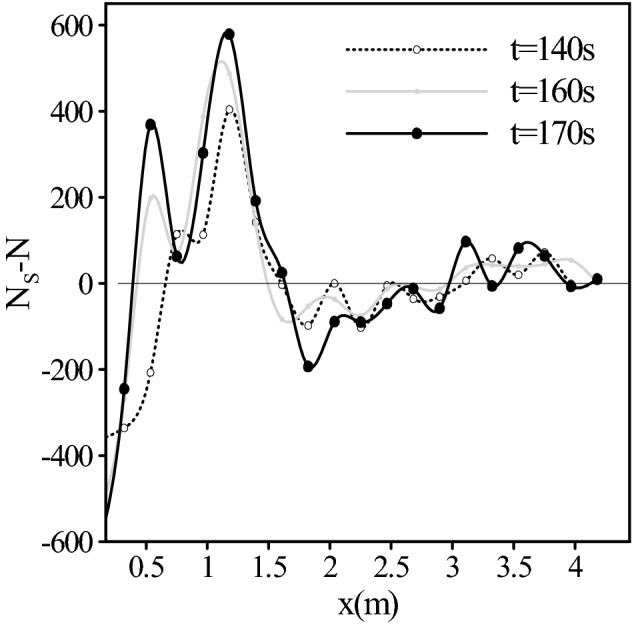
The changes in the pattern of sedimentation with temporal evolution.

Figure 15 is presented to get a better insight into the differences of deposition between two cases at different times which is focused on the discrepancy of the number of deposited particles in the cases with and without shear-induced lift force in different sections of channel, at different times.

It should be mentioned that the number of injected particles are the same for two cases in each second, but the difference in the pattern of sedimentation between the two cases increased in each second. As the high values of vorticity were generated steadily at the entrance of the channel as well as in the vicinity of the obstacle, it seems logical that the shear-induced lift force constantly altered the pattern of sedimentation. For example, between 140 and 170 s, the difference in the number of deposited particles at the channel entrance (0–0.2145 m) increased by 69.86%. In the regions with low values of vorticity (1.07–1.28 m), the difference in the number of deposited particles was increased by 43.31%. This behavior can be seen in the vicinity of the obstacle. For example, in the regions before the obstacle (1.7–1.9 m) the difference of deposited particles was incremented by 96.93%. In other words, the shear-induced lift force plays an important role in sedimentation. Without considering this force in such situations, the patterns of sedimentation can diverge from the real patterns through time.

### Coherent vortical structures

Q criterion was used to depict the nature of vortical structures of flow throughout the channel, This criterion shows the locations dominated by fluid rotation, consequently, we can visually determine vortex tubes.

The scalar Q is defined based on the second invariant of the velocity gradient tensor:

27 $$ {\text{Q}} = \frac{1}{2}(\Omega_{ij} \Omega_{ij} - S_{ij} S_{ij} ) $$

With $S_{ij}$ and $\Omega_{ij}$ denoting the symmetric and antisymmetric components of $\nabla u$ defined as:

28 $$ S_{ij} = \left( {\frac{{\partial u_{ij} }}{{\partial x_{ij} }} + \frac{{\partial u_{ij} }}{{\partial x_{ij} }}} \right) $$

29 $$ \Omega_{ij} = \left( {\frac{{\partial u_{ij} }}{{\partial x_{ij} }} - \frac{{\partial u_{ij} }}{{\partial x_{ij} }}} \right) $$

**Figure 16:**
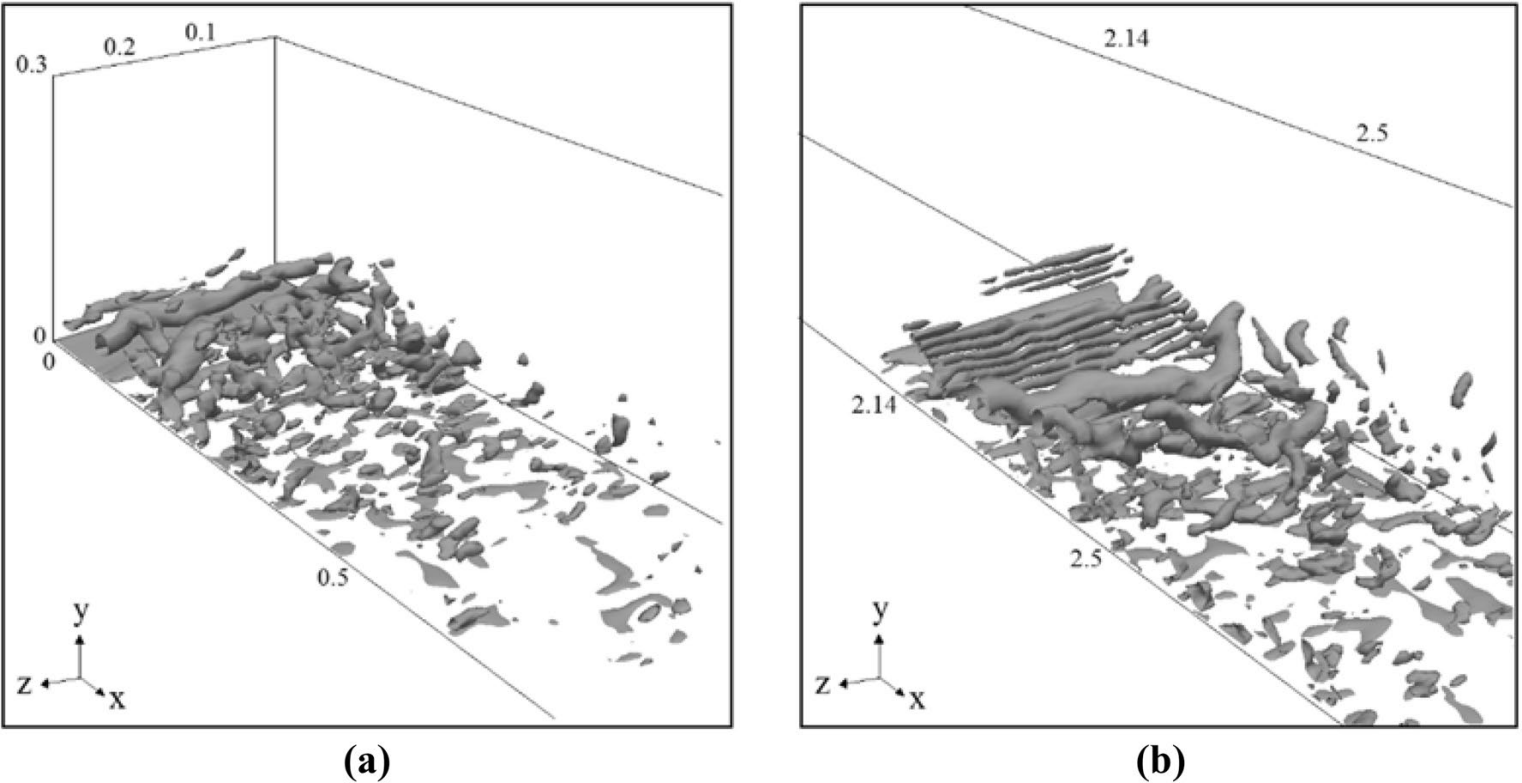
Vortical structures of gravity current at 150 s, (**a**) at the entrance, (**b**) after triangular obstacle.

In Fig. 16, strong vortical structures can be seen near the channel entrance and then their strength is decreasing along the channel. The existence of the obstacle enhanced vortical structures again. This is consistent with the values of vorticity shown in Fig. 12. We must keep in mind that in this paper the aim is to investigate the effect of shear-induced lift force in a particular condition in which the inlet condition and a triangular obstacle produce high-vorticities regions, but there are a wide range of conditions which can generate high values of vorticity (e.g. different shapes and heights of obstacle and inlet condition) and consequently, shear-induced lift force can have an important role in different conditions. For example, in Fig. 16a, the magnitude of vorticity and subsequently, the shape of vortical structures at the entrance of channel depend on the inlet condition. As the vortical structures are the result of velocity gradient, it seems logical that by increasing the inlet velocity, velocity gradients become higher due to no-slip condition on the bed of channel and it results in stronger vortical structures at the entrance.

An uncertainty analysis has been done in order to see the influence of the potentially important uncertainties on the results at the first 50 s, a time interval when we can identify the location of current head. The location of current head is a function of time and input parameters such as input velocity [0.11 m/s] and input concentration [0.9] and also the amount of deposition is related to the time and velocity. The total uncertainty of results is a function of uncertainties in the parameters as:

30 $$ \sigma_{h}^{2} = \left( {\frac{\partial h}{{\partial u}}} \right)^{2} \sigma_{u}^{2} + \left( {\frac{\partial h}{{\partial c}}} \right)^{2} \sigma_{c}^{2} $$

**Figure 17:**
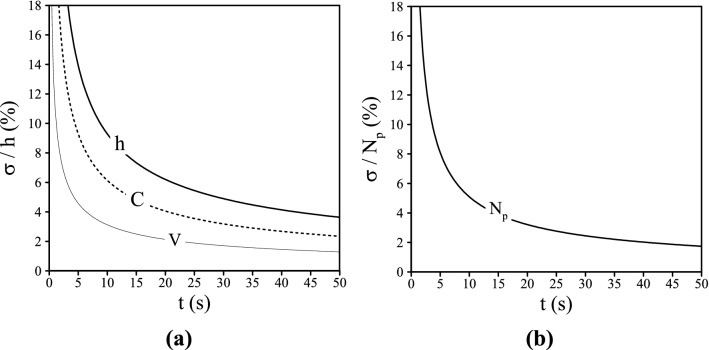
(**a**) The relative uncertainty of the current head (h) and the contributions of the input parameters (C = 0.9 ± 0.19% concentration and V: 0.11 ± 2.86%) as a function of time. (**b**) The relative uncertainty of the amount of particle deposition as a function of time and uncertainties in the inlet velocity V: 0.11 ± 2.86%.

$\sigma_{u}$ for velocity is 2.86% and $\sigma_{c}$ for concentration is 0.19%, based on standard deviation. Figure 17a presents the relative uncertainty of the current head and contributions of the uncertainties associated with input parameters (C: input concentration, V: input velocity) and Fig. 17b illustrates the relative uncertainty of the particles deposition as a function of uncertainty in the inlet velocity.

As shown in Fig. 17a the most relevant contribution is associated to concentration which is more than 65.5% of the total uncertainty of the current head. On the other hand, the contribution of the uncertainty in velocity is approximately 34.49% of the total uncertainty of the current head. Figure 17b shows that the relative uncertainty of particles deposition is decreasing over time. According to these results, it can be said that the input concentration is a more crucial factor than input velocity in order to control the head of current. Moreover, the input velocity is an important factor for controlling the amount of particles deposition specially in the first seconds of propagation.

## Conclusion

The Eulerian approach and LES model were employed to simulate turbulent gravity current, and Lagrangian markers were injected into the flow to explore the behavior of particles in the deposition process and to investigate the effect of shear-induced lift force (the Saffman force) on the sedimentation. The results showed that shear-induced lift force prevented particles from depositing at the channel entrance, in high-vorticity regions. For example, there was a reduction in the number of deposited particles by 1.75% in the first section of channel (0–0.2145 m) and 2.44% in the second section (0.2145–0.429 m) after 150 s. Consequently, under this condition, particles were transported by the current and deposited further at the downstream leading to an increase in the deposition rate in areas from 0.429 to 1.51 m by 1.172% after 150 s. Moreover, a reduction in the rate of sediment deposition can be seen again in the vicinity of obstacles due to the high values of vorticity which is generated near the triangular obstacle in the middle of channel. For instance, by applying shear-induced lift force the amount of deposited particles after the obstacle was 3.06% less than another case. The behavior of vorticity over a central line along the channel and values of vorticity in the vertical direction in some selected locations were illustrated in order to get a better insight into the behavior of shear-induced lift force in various sections of channel, which could depict the reason of changes in sedimentation profiles when shear-induced lift force was applied. For example, in the first section the vorticity reached a peak with a value of 67.87 1/s, where a reduction in deposition rate could be obviously seen. Q criterion was utilized to depict the vortical structures of flow. Vortical structures with a larger diameter indicated stronger vortices and were seen at the channel entrance, however, the vortex power decreased along the channel. The vortical structure of flow before the obstacle was not as strong as its condition at the entrance, but after the obstacle, especially the region near the obstacle, the vortical behavior of current intensified showing the role of an obstacle in vorticity generation. Deposition profiles in different times in cases with and without shear-induced lift force compared. The difference in the behavior of sedimentation increased over time. The important result is that if the shear-induced lift force on particles is neglected in the simulations, in the cases with high-vorticity regions in quasi-steady flows, the amounts of sedimentation will not be real.
